# Near field excited state imaging via stimulated electron energy gain spectroscopy of localized surface plasmon resonances in plasmonic nanorod antennas

**DOI:** 10.1038/s41598-020-69066-z

**Published:** 2020-07-27

**Authors:** Robyn Collette, David A. Garfinkel, Zhongwei Hu, David J. Masiello, Philip D. Rack

**Affiliations:** 1 Department of Materials Science and Engineering, University of Tennessee, Knoxville, TN 37996 USA; 2 Department of Chemistry, University of Washington, Seattle, WA 98195 USA; 3 Center for Nanophase Materials Science, Oak Ridge National Laboratory, Oak Ridge, TN 37831 USA

**Keywords:** Nanophotonics and plasmonics, Nanoparticles, Transmission electron microscopy

## Abstract

Continuous wave (cw) photon stimulated electron energy loss and gain spectroscopy (sEELS and sEEGS) is used to image the near field of optically stimulated localized surface plasmon resonance (LSPR) modes in nanorod antennas. An optical delivery system equipped with a nanomanipulator and a fiber-coupled laser diode is used to simultaneously irradiate plasmonic nanostructures in a (scanning) transmission electron microscope. The nanorod length is varied such that the *m* = 1, 2, and 3 LSPR modes are resonant with the laser energy and the optically stimulated near field spectra and images of these modes are measured. Various nanorod orientations are also investigated to explore retardation effects. Optical and electron beam simulations are used to rationalize the observed patterns. As expected, the odd modes are optically bright and result in observed sEEG responses. The *m* = 2 dark mode does not produce a sEEG response, however, when tilted such that retardation effects are operative, the sEEG signal emerges. Thus, we demonstrate that cw sEEGS is an effective tool in imaging the near field of the full set of nanorod plasmon modes of either parity.

## Introduction

The localized surface plasmon resonances (LSPR) sustained in noble metal nanostructures have inspired many new concepts in fields such as photovoltaics^1–3^, photocatalysis^4–6^, biosensing^7–9^, readout strategies for quantum computing^10,11^, and terahertz optical^12–14^ and magnetic meta atoms/materials^15–18^. While standard far field optical scattering techniques are used to probe the resonance conditions of individual nanostructures as well as nanostructure ensembles, probing the resultant near field is often more challenging. Several techniques such as scanning near field optical microscopy (SNOM)^19–23^, photoemission electron microscopy (PEEM)^24,25^, and electron energy loss spectroscopy (EELS)^26–29^ have been used to probe the near field distribution of LSPRs.

Of the near field techniques, EELS is unique in that the swift electron acts like a white (spectrally broad) evanescent field and thus can excite the full plasmonic spectrum of both bright and dark modes with atomic scale resolution. To this end, EELS has been utilized to characterize individual nanoparticle LSPRs as well as surface plasmon polaritons (SPP) and in particular the LSPR modes in nanorods^30–41^.

Beyond standard EELS, photoinduced near field electron microscopy (PINEM) is used to image the near field of optically excited nanostructures^42–47^. In PINEM, a pulsed laser photo-ejects electron beamlets or single electrons from the cathode, which are accelerated and arrive at the specimen synchronously to a second laser pulse that interacts with the sample. Thus PINEM enables the study of photoinduced near field phenomena at the nanoscale and the intense sample laser pulse (~ 1 × 10^15^ W/m^2^) induces photon stimulated electron energy loss (sEEL) and gain (sEEG) peaks. In addition to experimental demonstrations, several theoretical papers have described the sEEG and sEEL processes^48–51^. Additionally, by adjusting the timing of the cathode and sample laser pulse, temporal or so-called 4-dimensonal (x,y,z,t) information can be gleaned, which has been termed 4-dimensional (x,y,z,t) ultrafast electron microscopy/spectroscopy^52–56^. While interrogating temporal aspects reveals interesting physics, the PINEM instrument is quite complex and thus only a few instruments exist worldwide.

Recently, Das et al. demonstrated that by appropriately gating the EEL spectrometer, a high frequency nanosecond pulsed laser can be used to generate characteristic sEEL and sEEG with a continuous current electron source^57^. Furthermore, by coupling to a plasmonic nanostructure with a resonance at the laser frequency they demonstrated so-called resonant sEEL and sEEG. To further extend photoinduced electron microscopy and spectroscopy, we recently developed a laser system that can be installed on any (S)TEM system. Pulsed and continuous wave (cw) photothermal heating and excitation can both be achieved. In particular, we have studied the recrystallization, grain growth, phase separation, and dewetting of an Ag_0.5_Ni_0.5_ film^58^, and resonant cw sEEG and sEEL in nanostructures resulting from a dewet silver film^59^.

**Figure 1:**
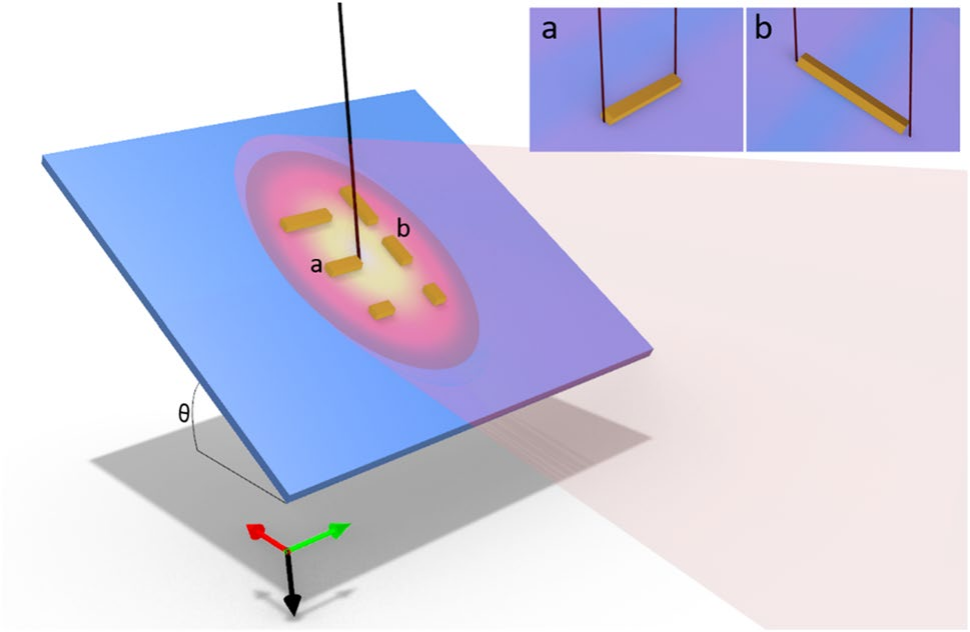
Experimental set up schematically illustrating the orientations of lithographically patterned gold nanorods aligned perpendicular and with a component parallel to the wave vector. Unpolarized light is directed toward the sample tilted at 30° (θ), thus the sample normal is oriented 30° to the electron beam trajectory and 60° to the photon wave vector. Inset shows magnified views of the nanorods illustrating the aloof positions for (**a**) horizontal nanorods (**b**) vertical nanorods and the electric field polarization component that couples to the LSPR modes.

Here we explore the cw photoexcited LSPR resonances of lithographically patterned gold nanorods with progressively longer lengths such that the *m* = 1, 2, and 3 longitudinal mode orders are resonant with the laser excitation energy (1.58 eV). As mentioned above, both even and odd parity LSPR modes are excited by the electron and revealed in EELS. Resonant sEEGS, however, requires far field coupling of the photons to the LSPR, thus it should be sensitive to the selection rules and retardation effects. The system (Fig. 1) is oriented such that the photon propagation and the electron beam propagation directions are perpendicular and oriented 60° and 30°, respectively, relative to the sample normal. Importantly the light is not polarized so all orientations can be excited as the electric field components aligned parallel to the longitudinal axis of the rods are selected by the rod antenna geometry. The nanorods are patterned such that the long axis is oriented with a component perpendicular (horizontal, Fig. 1a) and parallel (vertical, Fig. 1b) to the wave vector, thus we can control the s- and p-polarization of the light by tilting the sample and judiciously orienting the nanorods. Specifically, the electric field of the unpolarized light that couples to horizontally oriented rods are s-polarized, whereas the electric field of light that couples to vertical rods have a mixed s- and p-polarized component; thus, the vertical rods conveniently enables us to compare retardation effects in sEEGS.

## Experimental section

1. Sample Fabrication
  Au nanorods with various dimensions and orientations (see Table 1) and 60 nm thickness were patterned on a 30 nm thick Si_3_N_4_ TEM membrane using electron beam lithography. Nanorod lengths were chosen such that the plasmon modes (*m* = 1, 2 and 3) are resonant near the 1.58 eV laser photon energy.
2. EEL and EEG measurements
  A Zeiss Libra TEM was operated at an accelerating voltage of 200 kV in (S)TEM. The camera length is set to 378 mm. The collection semiangle (β) is 100 mrad and convergence semiangle (α) was 0 mrad. A monochromator slit of 0.5 µm is used for spectrum acquisition with the dispersion set as 30 meV per channel. EELS map acquisition details are summarized in Table 1. Maps are generated using the Gatan Digital Micrograph spectra by plotting spectra intensity for specific energy slices from the 3D spectrum image data cube. Low-loss point spectrum acquisition details are summarized in Table 1. Low-loss point spectra are post processed by aligning the zero-loss peak to 0 eV, followed by normalizing to the integrated number of counts and dividing by the channel resolution. The sample is irradiated with a fiber-coupled 1.58 eV laser diode with tunable optical power up to 215 mW focused to ~ 5 µm diameter. The sample is tilted at 30° and the unpolarized Gaussian laser spot is aligned and focused to the coincident (S)TEM electron point (see^58^ for system details). The laser is operated in cw mode at 1.01 × 10^9^ W/m^2^ for all laser-on results presented here. Maps and individual point spectra were acquired with the laser off and with the laser on to observe the resonant sEEL and sEEG peaks.

**Table 1:** Map collection data, point spectra collection data, nanorod dimensions, and corresponding relevant mode resonance for *m* = 1, 2, and 3 nanorods. Subscript indicates orientation (horizontal or vertical) and laser condition (on or off).

	Map pixel time (s)	Map pixel size (nm)	Spectra frames	Spectra exposure (s)	Nanorod length (nm)	Nanorod width (nm)	Peak (eV)
*m* = 1 _H, On_	0.05	10 × 11.55	10	0.05	180	71	1.62
*m* = 1 _H, Off_	0.05	11 × 12.70	5	0.05			
*m* = 1 V_, On_	0.065	7.9 × 9.12	10	0.06	150	63	1.53
*m* = 1 V_, Off_	0.065	7.5 × 8.66	5	0.065			
*m* = 2 _H, On_	0.05	9.1 × 10.51	10	0.05	330	71	1.66
*m* = 2 _H, Off_	0.05	9.1 × 10.51	5	0.05			
*m* = 2 V_, On_	0.065	10 × 11.55	6	0.065	310	52	1.54
*m* = 2 V_, Off_	0.065	9.4 × 10.85	5	0.06			
*m* = 3 _H, On_	0.05	15 × 17.32	10	0.05	670	82	1.49
*m* = 3 _H, Off_	0.05	16 × 18.48	5	0.05			
*m* = 3 V_, On_	0.05	18 × 20.78	5	0.05	660	83	1.46
*m* = 3 V_, Off_	0.05	18 × 20.78	5	0.05			

## Results

**Figure 2:**
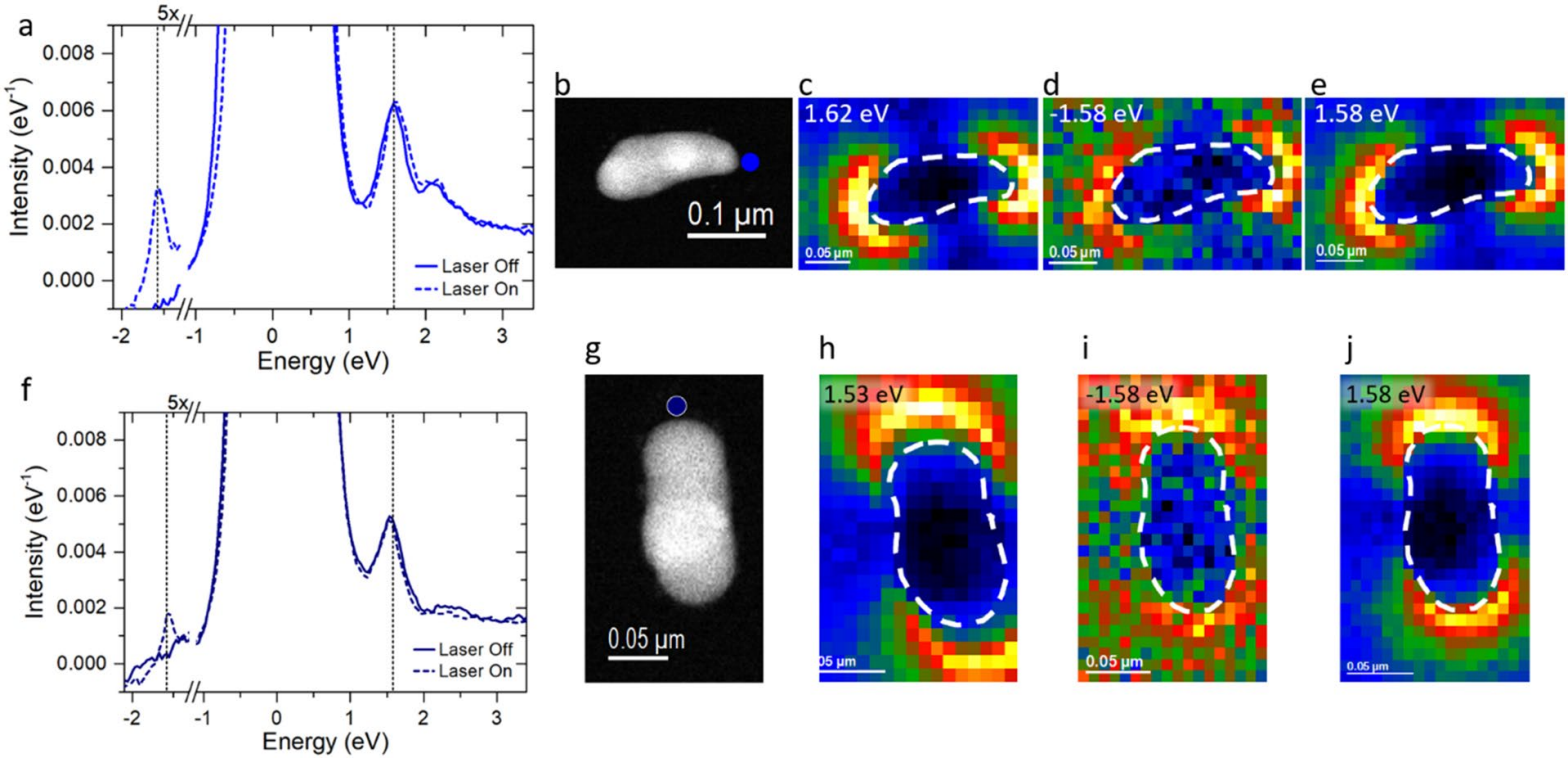
(**a**) 180 nm horizontal nanorod aloof 6-pixel map spectra average with laser off and on. Dashed lines in a and f correspond to the laser energy at ± 1.58 eV (**b**) HAADF image of horizontal nanorod with aloof position indicated by blue circle. (**c**–**e**) Horizontal nanorod maps of EEL, sEEG, and sEEL peak intensities, respectively. (**f**) 150 nm vertical nanorod aloof 6-pixel map spectra average with laser off and on. (**g**) HAADF image of vertical nanorod with aloof position indicated by blue circle. (**h**–**j**) Vertical nanorod maps of EEL, sEEG, and sEEL peak intensities, respectively.

First, we probe the optically bright *m* = 1 or dipolar LSPR mode. Figure 2a displays the EEL/G point spectra of a ~ 180 nm horizontal nanorod collected at the aloof position at one of the long axis ends with and without concurrent laser irradiation (see Fig. 2b for nanorod image and position). The nanorod long axis is perpendicular to the photon propagation direction and thus only s-polarized light couples with the nanorod. The laser-off spectrum is taken for reference and is excited by the high energy electron beam, which conveniently couples to both bright and dark plasmons and reveals the full plasmonic spectrum. The laser-off spectrum has a dipole resonance at 1.62 eV and a peak at 2.25 eV, which is attributed to the higher order LSPR modes. The laser-off 1.62 eV EELS map is shown in Fig. 2c, which has the expected intensity peaks at the nanorod ends (see supplement for complementary map at 2.25 eV). The laser-on EEL point spectrum is similar to the laser-off spectrum except a small sEEL peak and sEEG peak emerges at ± 1.58 eV, respectively. The laser-on EELS map is shown in Fig. 2 for − 1.58 (d) and + 1.58 eV (e). Clearly, the sEEG and sEEL peaks have the signature dipolar characteristics and thus the photons are resonantly coupling to the dipole or *m* = 1 LSPR mode.

The 150 nm vertical nanorod is oriented such that the long axis has a component parallel to the photon propagation direction thus both s- and p-polarized light couples with the long axis dipole that is resonant with the laser energy. The EEL point spectra for the laser-on and laser-off condition of the vertical nanorod are plotted in Fig. 2f for the aloof position in Fig. 2g. The dipole resonance of this nanorod is ~ 1.53 eV and the higher order modes at 2.43 eV. Figure 2h shows the 1.53 eV EELS map. For the laser-on spectrum, the photon-plasmon coupling is again evidenced via the emergence of the sEEL and sEEG peaks at ± 1.58 eV. Figure 2 shows the laser-on maps of the sEEL (i) and sEEG (j) peaks. As will be discussed below, the tilted orientation slightly decreases the spontaneous EELS intensity and the s-polarized component of the polarized light that is aligned with the long axis is reduced due to the orientation; thus the sEEL/sEEG intensity is reduced relative to the horizontal orientation^60^.

The *m* = 2 mode is interrogated using longer nanorods of ~ 310 (vertical) and 330 nm (horizontal) in length. In contrast to optical techniques, an electron beam is capable of exciting all plasmonic modes, thus we expect to observe an EEL signature related to the *m* = 2 mode. However, the sEEG and sEEL signatures are produced by synergistic electron and optical coupling and because this mode is optically dark, no sEEL and sEEG peaks should appear. However, as will be shown, appropriate orientations induce retardation effects^35,61,62^, which enhance the far field photon coupling and the emergence of resonant sEEL and sEEG peaks.

**Figure 3:**
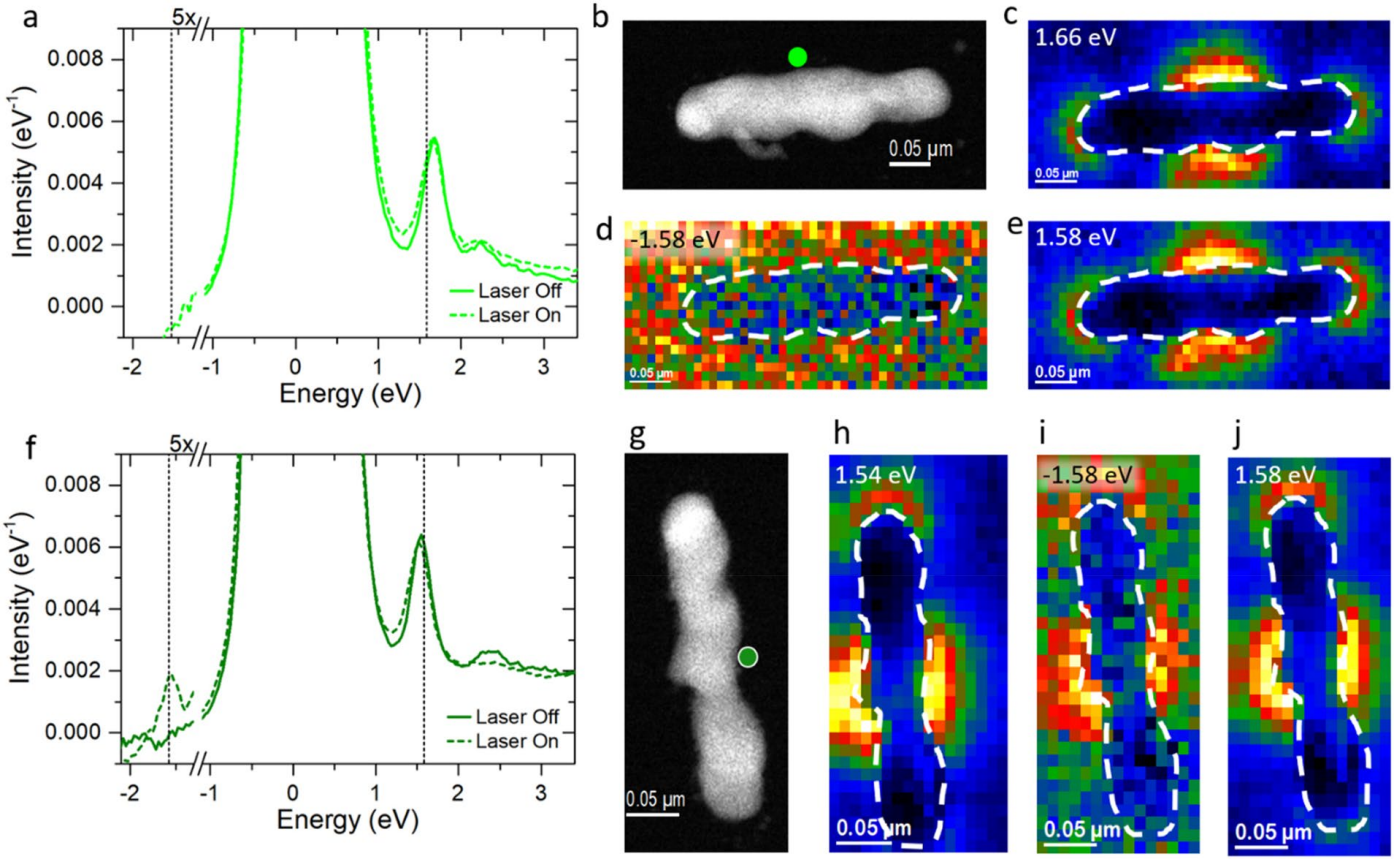
(**a**) 330 nm horizontal nanorod aloof 6-pixel map spectra average with laser off and on. Dashed lines in a and f correspond to the laser energy at ± 1.58 eV. (**b**) HAADF image of horizontal nanorod with aloof position indicated by green circle. (**c**–**e**) Horizontal nanorod maps of EEL, sEEG, and sEEL peak intensities, respectively. (**f**) 310 nm vertical nanorod aloof 6-pixel map spectra average with laser off and on. (**g**) HAADF image of vertical nanorod with aloof position indicated by green circle. (**h**–**j**) Vertical nanorod maps of EEL, sEEG, and sEEL peak intensities, respectively.

Figure 3a displays the EEL point spectra of the ~ 330 nm horizontal long nanorod collected at the long axis center aloof position with and without concurrent laser irradiation (Fig. 3b). The laser-off spectrum has peaks at 1.66 eV and at 2.35 eV, which are attributed to the *m* = 2 mode and the higher order modes, respectively. The laser-off 1.66 eV EELS map is shown in Fig. 3c, which reveals the expected peak intensity on each nanorod end and in the nanorod center, where the loss probability is the highest. Additionally, the ZLP appears narrower when taken at the nanorod center than the spectra collected at the nanorod ends because the low-energy dipole resonance broadens the ZLP (see SI). The laser-on EEL point spectrum is very similar to the laser-off EELS spectrum. The ZLP is slightly broadened due to photothermal heating, however, no sEEL or sEEG peaks are observed at ± 1.58 eV, respectively. Figure 3d shows the EELS map for − 1.58 eV, which does not contain the signature of the *m* = 2 pattern. Figure 3e shows the EELS map for + 1.58 eV, which demonstrates the *m* = 2 mode, however this is due to the spontaneous EEL and not sEEL. Thus, clearly there is no optical coupling observed.

The 310 nm m = 2 vertical nanorod EEL point spectra for the laser-off and laser-on conditions are plotted in Fig. 3f for the center aloof position (Fig. 3g). The *m* = 2 resonance for this nanorod occurs at 1.54 eV and the higher order modes are 2.46 eV. Figure 3h shows the 1.54 eV EELS map, where unexpectedly the mode signature is more intense at the top of the nanorod relative to the bottom. As expected, due to retardation effects, the laser-on spectrum clearly possesses the sEEG peak at − 1.58 eV. Figure 3i,j show the sEEG and sEEL maps, which clearly exhibit the characteristic *m* = 2 intensity profile. The selection rules for optical coupling are relaxed due to the geometry of our experiment. It is known that optically dark modes can be excited by using an oblique angle of incidence of light, which introduces phase retardation across a structure^35,61,62^. When the long axis of the nanorod is oriented with a component parallel to the photon propagation axis (p-polarization), as it is for our vertical orientation, retardation effects are induced where the strength of the electric field is non-uniform along the nanorod long axis, thus allowing for optical excitation of the *m* = 2 mode^60^. In the case of the horizontal nanorod, the long axis is perpendicular to the light propagation, resulting in no phase retardation, and the *m* = 2 resonance sEEL and sEEG peaks are not observed.

**Figure 4:**
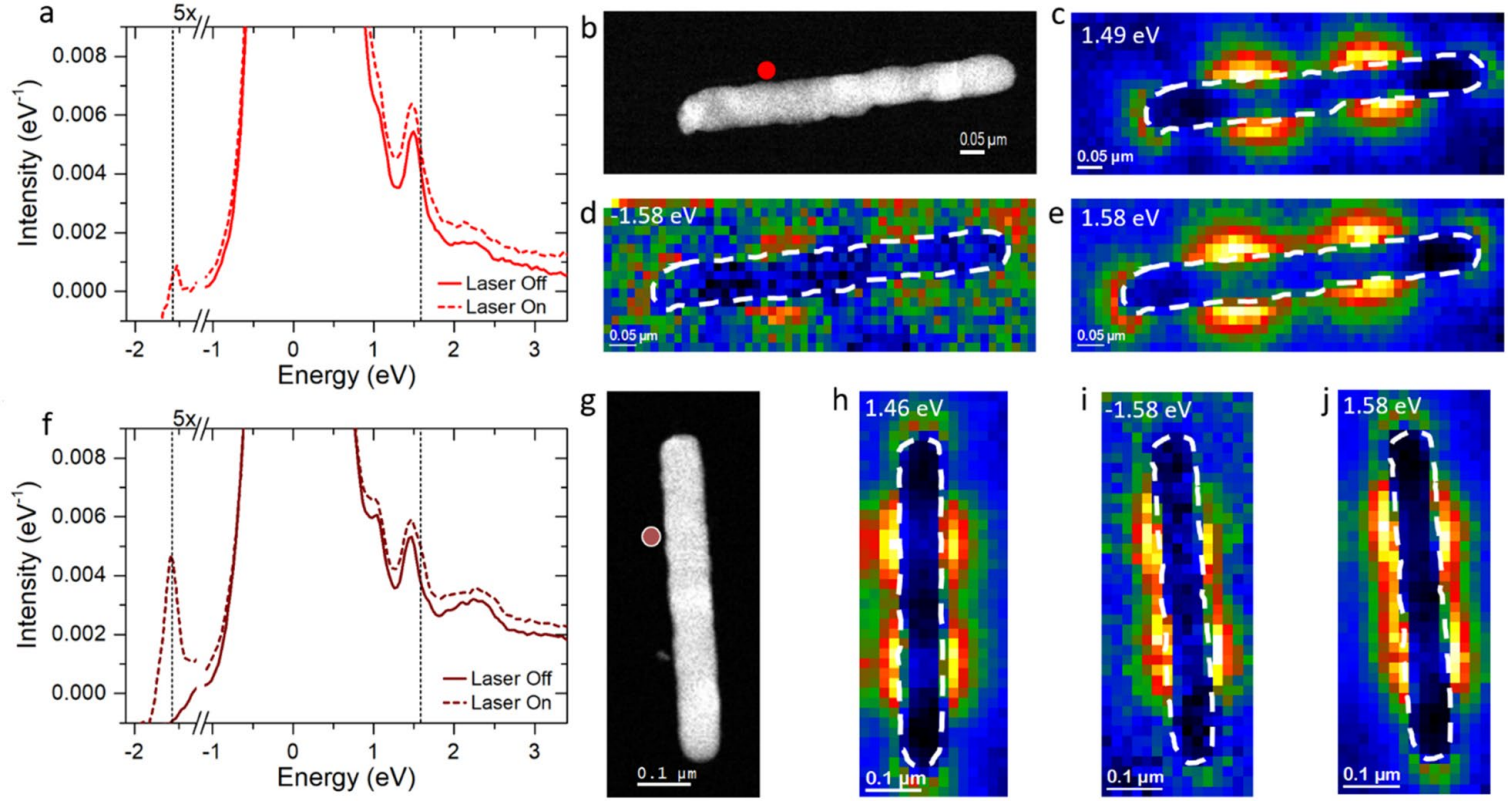
(**a**) 670 nm horizontal nanorod aloof 6-pixel map spectra average with laser off and on. Dashed lines in (**a**) and (**f**) correspond to the laser energy at ± 1.58 eV. (**b**) HAADF image of horizontal nanorod with aloof position indicated by red circle. (**c**–**e**) Horizontal nanorod maps of EEL, sEEG, and sEEL peak intensities, respectively. (**f**) 660 nm vertical nanorod aloof 6-pixel map spectra average with laser on. (**g**) HAADF image of vertical nanorod with aloof position indicated by red circle. (**h**–**j**) Vertical nanorod maps of EEL, sEEG, and sEEL peak intensities, respectively.

The *m* = 3 mode is probed to investigate coupling to higher order bright modes. Figure 4a displays the EEL point spectra of the ~ 670 nm horizontal nanorod collected at an aloof position 1/3 the length of the nanorod (Fig. 4b) where the *m* = 3 mode is expected to have the strongest resonance. The laser-off spectrum shows a resonance at 1.49 eV and the 1.49 eV EELS map is shown in Fig. 4c, which clearly demonstrates the EELS intensity peaks at the 1/3 and 2/3 rod length positions associated with the *m* = 3 mode. Additionally, the *m* = 2 mode is observed as a shoulder to the ZLP (see SI for *m* = 2 map). The laser-on point spectrum shows the characteristic sEEL and sEEG peaks at ± 1.58 eV. Figure 4d,e show the EELS maps for the sEEG and sEEL energies, which also clearly have the characteristic *m* = 3 nodal pattern, demonstrating resonant coupling to the *m* = 3 mode.

The 660 nm vertical nanorod point EEL spectra are shown in Fig. 4f for aloof position indicated in Fig. 4g. Here, we see a peak at 1.46 eV which is attributed to the *m* = 3 mode as evidenced by the EELS map in Fig. 4h. The laser-on point spectrum shows the characteristic sEEL and sEEG peaks at ± 1.58 eV. The EELS maps for the sEEG and sEEL energies are shown in Fig. 4i,j, which demonstrate the *m* = 3 pattern.

## Discussion

**Figure 5:**
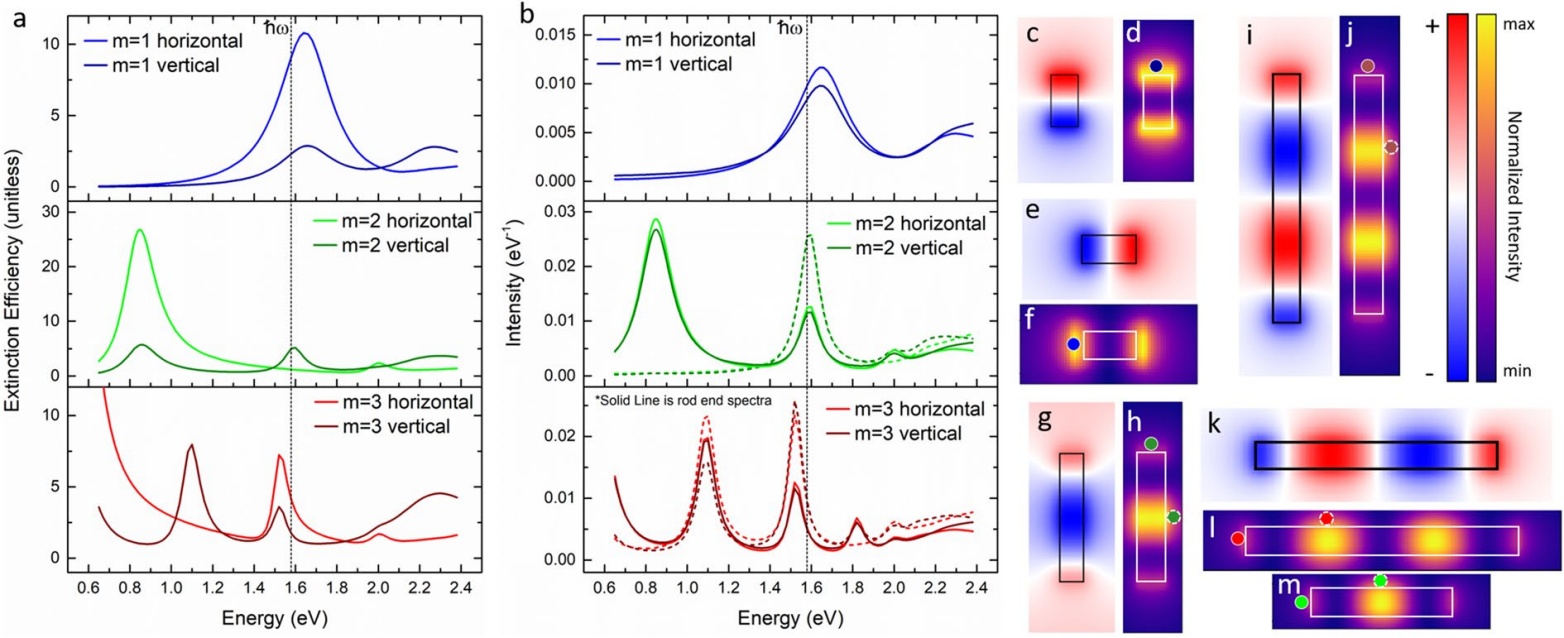
Simulated extinction (**a**) and EEL (**b**) spectra for horizontal and vertical rod orientations where the EELS are taken at a common 9 nm impact position relative to the nanorod end. Positions indicated in EEL maps by color coded circles with solid white boarder for spectra taken at the end of the nanorod and with a dashed white boarder for spectra taken at the EELS intensity maximum for *m* = 2, 3. Normalized extinction (**c**, **e**, **g**, **i**, **k**, **m**) and EEL (**d**, **f**, **h**, **j**, **l**) maps for the *m* = 1 (**c**–**f**), *m* = 2 (**g**, **h**, **m**), and *m* = 3 (**i**–**l**) modes of the two nanorod orientations for the experimental geometries.

Several approaches have been developed to model photon stimulated EEL and EEG phenomena^48–51,57,59^. As has been demonstrated previously^59^, sEELS and sEEGS is approximately proportional to the product of the optical extinction cross section (σ) and the spontaneous EELS intensity (Γ_EELS_). Thus it is instructive to compare the resultant EEL and extinction spectra for the geometries studied. We performed discrete-dipole approximation (DDA)^63,64^ and electron-driven DDA (e-DDA)^65,66^ simulations of the different nanorod lengths. Figure 5 shows DDA electric field maps (c, e, g, i, k) and EELS maps (d, f, h, j, l, m) for the *m* = 1 (c-f), *m* = 2 (g, h, m) and *m* = 3 (i–l) of the two nanorod orientations. Figure 5a,b are the simulated extinction and EEL spectra, taken at a 9 nm impact parameter at the intensity maximum in the EELS map for each rod (see simulated EELS maps for spectral positions). As illustrated in Fig. 1, the electron beam trajectory is 30° and the photon wave vector is 60° relative to the nanorod normal.

Before overviewing the sEEGS results, it is worth noting a few general observations of the EEL and extinction spectra for the two orientations (see supplemental information for details). First, regarding the EEL spectra, note that the tilted substrate slightly decreases the Γ_EELS_ for the end position in the vertical orientation, whereas it has a negligible effect on the horizontal orientation. Additionally, when the long nanorod axis is in the vertical orientation, the component of the electric field polarization that couples with the long axis is decreased due to the tilt by sin^2^(30°) or 25%; the horizontal orientation, however, is constant. With these generalizations, we expect that for the odd bright modes (*m* = 1 and 3), the sEEG of the horizontal orientations should be more intense as both the electric field and Γ_EELS_ are higher. Interestingly, for the *m* = 3 mode the Γ_EELS_ is ~ 2 × higher than the *m* = 1 mode, but the optical extinction is ~ 2 × lower so the sEEG intensity should be comparable.

As noted previously^51^ and confirmed in our previous work^59^, the sEELS and sEEGS peaks have nearly the same amplitude and thus while the sEEL peaks are convolved with the LSPR peaks, we can unambiguously fit the sEEG peaks and thus de-convolve the sEEL and LSPR peaks (see supplemental information for peak fitting). Furthermore, Das et al.^57^ showed that the light-driven population of the plasmon mode (M_x_^max^) can be estimated by $M_{x}^{max} = \left[ {\left( {\frac{{{\Gamma }_{EEL} + {\Gamma }_{sEEL} }}{{{\Gamma }_{sEEG} }}} \right) - 1} \right]^{ - 1}$ ; where Γ_EEL_, Γ_sEEL_, and Γ_sEEG_, are the integrated peak intensities of spontaneous EELS and the associated sEEL and sEEG peaks of the SPP mode of interest. Table 2 summarizes the M_x_^max^ numbers estimated from the peak fits of the spectra taken at the spontaneous EELS intensity maximum positions for each mode. Note that while higher light-driven plasmon populations are realized in high-irradiance pulsed experiments^57^, the values realized here are consistent with previous low irradiance cw experiments^59^.

**Table 2:** Light driven plasmon populations (M_x_^max^).

	Horizontal	Vertical
*m* = 1	6.1 × 10^–2^	1.7 × 10^–2^
*m* = 2	–	6.3 × 10^–2^
*m* = 3	7.3 × 10^–2^	1.63 × 10^–1^

Empirically, the light-driven population is proportional to the laser irradiance and the extinction coefficient at the laser energy. Assuming the laser irradiance is constant, one can compare the experimental M_x_^max^ values to the calculated extinction coefficients of the different modes and orientations. As expected, the horizontal *m* = 1 plasmon occupation stimulated by the laser is 3.6 × the vertical nanorod in excellent agreement with the 4 × reduction expected from the reduced electric field for the vertical orientation. Interestingly, light-driven plasmon population for the horizontal *m* = 3 is slightly higher than the *m* = 1, though one expects that the optical coupling to the *m* = 1 mode would be ~ 2 × that of the *m* = 3 value. Even more surprisingly, the vertical *m* = 3 light driven plasmon population has the highest value, which is > 2 × greater than the *m* = 1 horizontal dipole, which has a simulated extinction cross section 5 × smaller. Small variations in the alignment of the Gaussian laser profile, variations in the impact parameters, and perhaps geometric asymmetries present in the nanorod could be contributing factors to some of the quantitative inconsistencies. We note that the *m* = 3 rods are the most regular patterns and have much less roughness, which could enhance the dephasing time relative to the m = 1,2 modes.

For the *m* = 2 dark mode in the horizontal orientation, the extinction cross section is near zero and thus no optical coupling or sEEG is observed. For the vertical orientation, however, the mixed s- and p-polarization induces retardation effects, which increases the extinction cross section and thus the sEEG peak emerges. While the simulated extinction cross section ratio *m* = 2(vertical)/*m* = 1(horizontal) is ~ 0.5, the experimental light driven plasmon population ratio is ~ 1. Similarly, the simulated extinction cross section *m* = 2(vertical)/*m* = 1(vertical) is ~ 2.1 and the experimental light driven plasmon population ratio is 3.7. Interestingly, there is a competition in the extinction cross section for the *m* = 2 mode as a function of the sample tilt angle; starting at θ = 90° and as θ→0, retardation enhances the extinction cross section, however the electric field decreases. The result is that the extinction efficiency for this mode is a maximum at 45°. Thus, a judicious use of laser orientation and/or substrate tilt can be used to promote sEEGS as a unique tool to observe the near field of optically excited materials.

## Conclusions

We have shown that continuous wave (cw) photon stimulated electron energy loss and gain spectroscopy can be used to image the near field of optically stimulated LSPR modes in nanorod antennas. The sEEL and sEEG peaks are generated by an optical delivery system mounted on a (S)TEM microscope. The LSPR *m* = 1, 2, and 3 modes are tuned to the laser energy by varying the nanorod length. The optically stimulated near field spectra and images of these modes are measured at various nanorod orientations to explore how the electric field and retardation affect the resonant sEEG. By fitting the spectra and obtaining the integrated peak intensities of spontaneous EEL and the associated sEEL and sEEG peaks, we estimated the light-driven population of the plasmon mode for each nanorod. DDA and e-DDA simulations of the extinction coefficients and EEL probabilities, respectively, are used to rationalize the observed data. As expected, the odd modes are optically bright and thus sEEG peaks are observed. The *m* = 2 dark mode promotes sEEG only when oriented vertically and tilted such that mixed s-and p-polarization induced retardation effects and thus increase the extinction coefficient of this mode. Thus, we demonstrate cw sEEGS as an effective tool in imaging the near field of optically driven plasmon modes.

## Supplementary information

Supplementary Information.
