# Supplementary material for: Near field excited state imaging via stimulated electron energy gain spectroscopy of localized surface plasmon resonances in plasmonic nanorod antennas
Source: Sci Rep. 2020 Jul 27;10:12537. doi: 10.1038/s41598-020-69066-z (PMC7385139; doi:10.1038/s41598-020-69066-z)
Supplement: Supplementary file 1 — Supplementary Information. [file 41598_2020_69066_MOESM1_ESM.pdf]

Supporting Information

Near Field Excited State Imaging via Stimulated Electron Energy Gain Spectroscopy of Localized Surface Plasmon Resonances in Plasmonic Nanorod Antennas

Robyn Collette[a], David A. Garfinkel[a], Zhongwei Hu[b], David J. Masiello[b], and Philip D. Rack[a,c*]

[a] Department of Materials Science and Engineering, University of Tennessee, Knoxville, Tennessee 37996, United States

[b] Department of Chemistry, University of Washington, Seattle, Washington 98195, USA

[c] Center for Nanophase Materials Science, Oak Ridge National Laboratory, Oak Ridge, Tennessee 37831, United States

* Corresponding Author: prack@utk.edu

Supplementary Table S1. Point spectra collection parameters.

	Left/Top		Center		Right/Bottom	
	**Frames**	**Exposure**	**Frames**	**Exposure**	**Frames**	**Exposure**
**M1** $_{H, On}$	10	0.05	-	-	10	0.05
**M1** $_{H, Off}$	5	0.05	-	-	5	0.05
**M1** $_{V, On}$	5	0.06	-	-	10	0.06
**M1** $_{V, Off}$	5	0.065	-	-	5	0.065
**M2** $_{H, On}$	10	0.05	10	0.05	10	0.05
**M2** $_{H, Off}$	5	0.05	5	0.05	5	0.05
**M2** $_{V, On}$	10	0.065	6	0.065	10	0.065
**M2** $_{V, Off}$	6	0.06	5	0.06	7	0.06
**M3** $_{H, On}$	10	0.05	10	0.05	10	0.05
**M3** $_{H, Off}$	5	0.05	5	0.05	5	0.05
**M3** $_{V, On}$	5	0.06	5	0.06	5	0.06
**M3** $_{V, Off}$	5	0.06	5	0.06	5	0.06

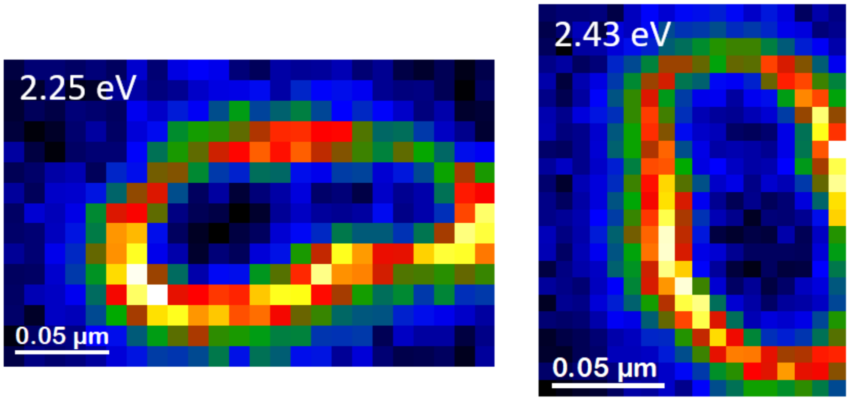

Supplementary Figure S1. Maps of the higher order modes for horizontal and vertical rods from m=1 length rods.

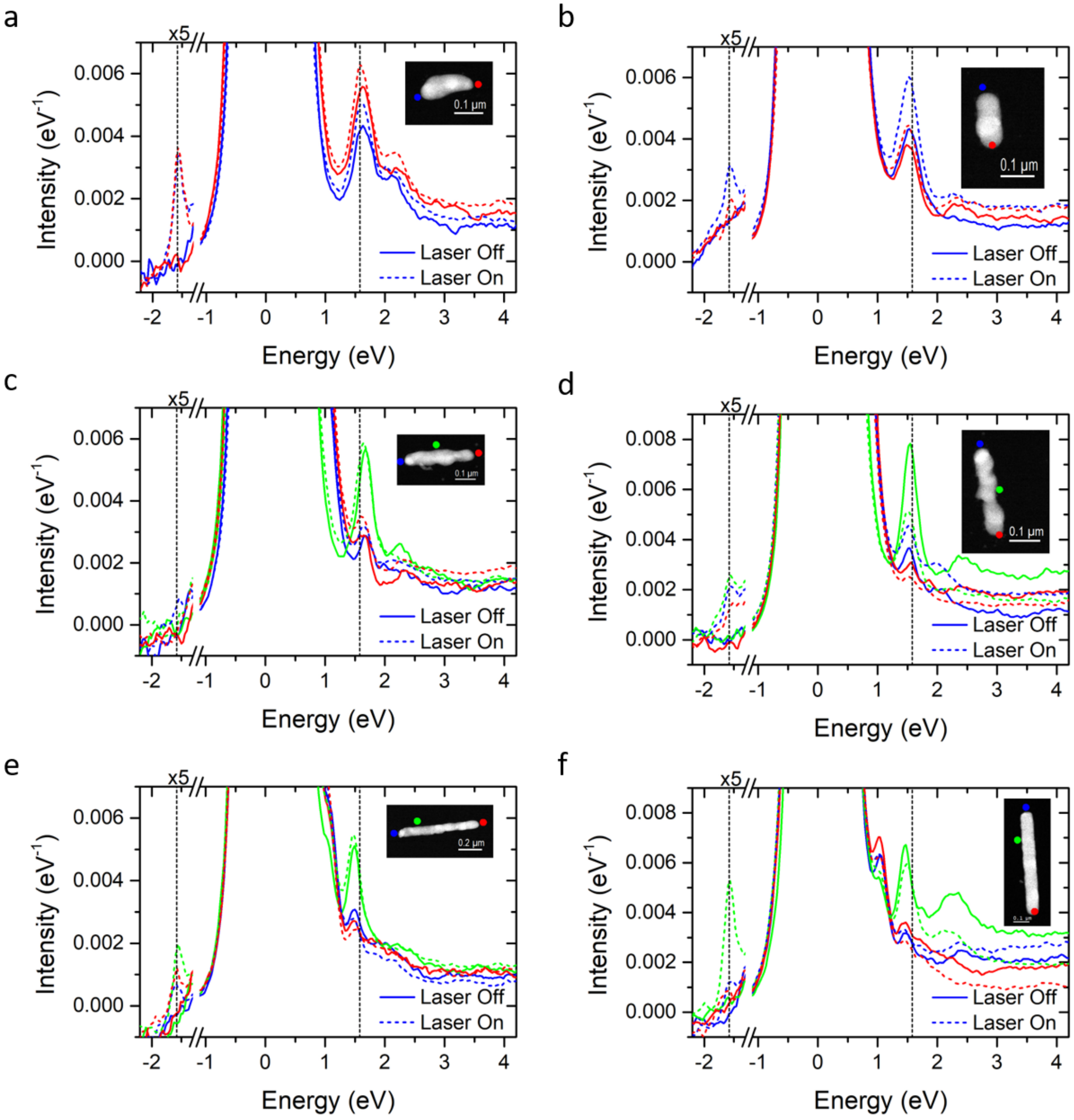

Supplementary Figure S2. Point spectra for laser off and laser on conditions for (a) horizontal m=1 mode rods, (b) vertical m=1 rods, (c) horizontal m=2 rods, (d) vertical m=2 rods, (e) horizontal m=3 rods, (f) vertical m=3 rods with HAADF images and aloof positions show in the inset.

SI – Fitting

In order to quantify the number of plasmons, we fit the unprocessed experimental spectra. An example of the fitting for the *m*=1 horizontal rod is shown in Supplementary Figure S3. We employ the built-in *Matlab* algorithm, *fmincon*, using the chi-squared distribution function as the objective function to find the best fit. Prior to fitting, the origin was determined by fitting zero-loss peak to a Gaussian function and shifted the peak to zero eV to ensure the exact positions of peaks in the spectrum. The intensity of each spectrum was normalized by the integrated sum. The zero-loss peak was fit with a gaussian and two exponential tails, one on the positive and one on the negative energy side of the zero-loss peak. Upon subtraction of the zero-loss peak, four Gaussian peaks were used to model the Si_3N_4 substrate, and the remaining peaks were fit using Lorentzian peaks. The whole spectrum was fit using the minimum number of peaks required for a good fit.

Fitting parameters for the substrate and five rods are shown in Supplementary Table S2-Table S7. A spectrum acquired far from any resonant structures was used to first fit the substrate signal. The large broad peak around 22 eV is from the bulk plasmon of the Si_3N_4 substrate, which was best represented by four Gaussian peaks (Supplementary Table S2). The parameters found for these four peaks were allowed to change by 10% for subsequent fitting of spectra acquired for resonant structures. Extra peaks are seen around the zero-loss as was previously reported[1]. The laser off spectrum was first fit using the minimum number of peaks. To fit the laser on spectrum, two identical peaks were added for the sEEG (-1.58 eV) and sEEL (1.58 eV). The sEEG/sEEL peaks were set to have the same half-width-at-half-maximum (HWHM) as the zero-loss peak and the amplitude was unrestricted. The plasmon peak (*m*=1,2,3) amplitudes were restricted to be within 10% of the laser off spectrum since the addition of the sEEL peak allows for multiple representations of this area of the spectrum. The additional peaks from the laser off spectrum were used as initial conditions where the position and HWHM were allowed to vary by 10% and the amplitude was unrestricted. The resultant peak fitting parameters are listed in the tables below.

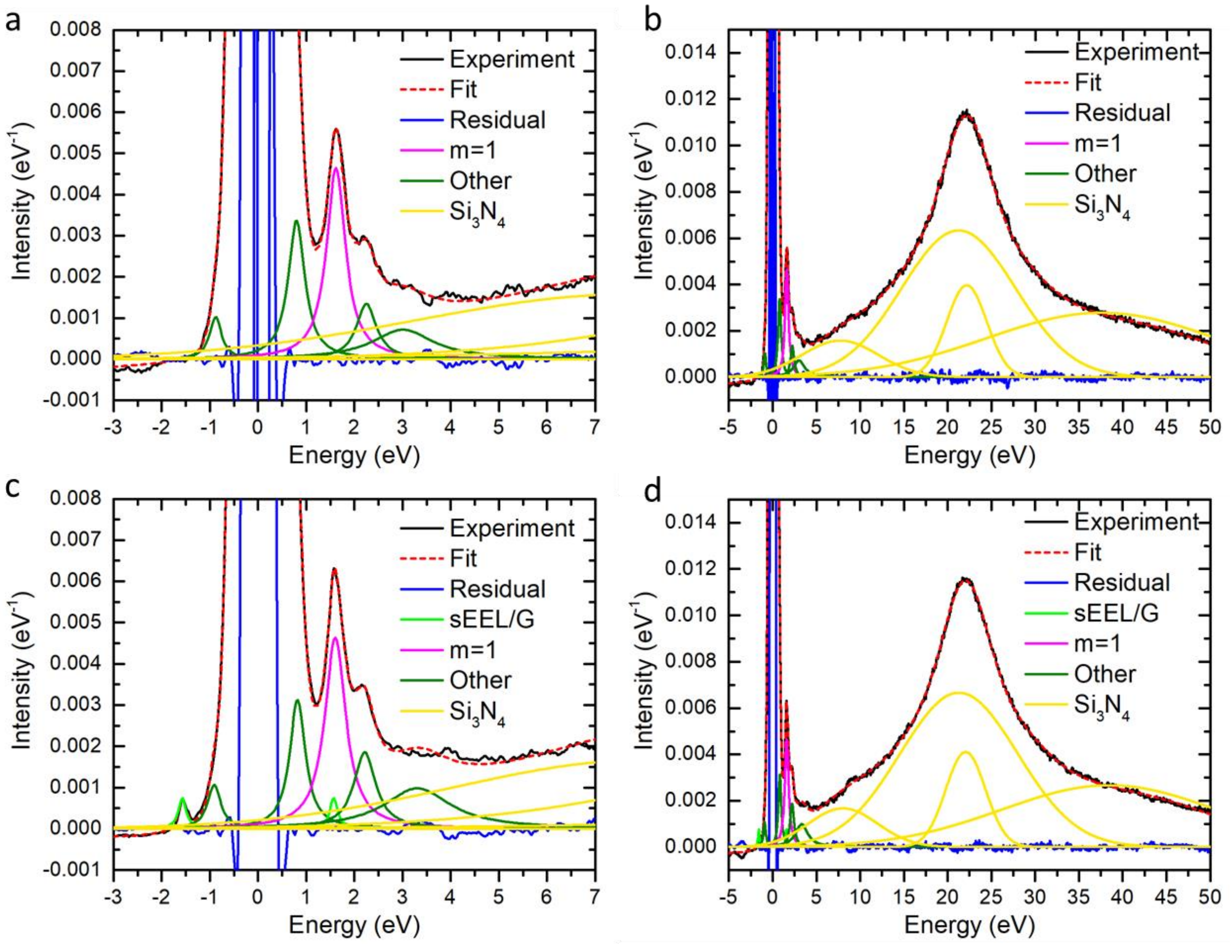

Supplementary Figure S3. Representative fits for horizontal m=1 rod for laser off (a-b) and laser on (c-d) spectra.

Supplementary Table S2. Fitting parameters for Si_3N_4 substrate.

	Laser Off		
	**Amplitude (eV^{-1})**	**Center (eV)**	**HWHM (eV)**
**Substrate**	0.00564	21.436	7.520
**Substrate**	0.00423	22.035	2.821
**Substrate**	0.00276	36.305	17.201
**Substrate**	0.00110	9.616	4.804

Supplementary Table S3. Fitting parameters for horizontal m=1 rod for laser off and laser on spectra.

	Laser Off			Laser On		
	**Amplitude (eV^{-1})**	**Center (eV)**	**HWHM (eV)**	**Amplitude (eV^{-1})**	**Center (eV)**	**HWHM (eV)**
**sEEG**	-	-	-	0.00075	-1.572	0.099
**Thermal**	0.00102	-0.880	0.151	0.00106	-0.910	0.165
**Thermal**	0.00337	0.805	0.212	0.00313	0.821	0.198
**sEEL**	-	-	-	0.00075	1.573	0.099
**m=1**	0.00464	1.623	0.242	0.00464	1.605	0.264
**Higher Order**	0.00135	2.254	0.255	0.00186	2.217	0.275
	0.00072	3.011	0.770	0.00098	3.311	0.847
**Substrate**	0.00634	21.227	7.650	0.00666	21.283	7.905
**Substrate**	0.00396	22.158	2.688	0.00410	22.062	2.680
**Substrate**	0.00278	37.548	15.397	0.00266	38.447	14.407
**Substrate**	0.00158	7.707	5.156	0.00167	8.029	4.644

Supplementary Table S4. Fitting parameters for vertical m=1 rod for laser off and laser on spectra.

	Laser Off			Laser On		
	**Amplitude (eV^{-1})**	**Center (eV)**	**HWHM (eV)**	**Amplitude (eV^{-1})**	**Center (eV)**	**HWHM (eV)**
**sEEG**	-	-	-	0.00019	-1.572	0.093
**Thermal**	0.00050	-1.018	0.168	0.00092	-0.918	0.176
**Thermal**	0.00254	0.850	0.253	0.00249	0.844	0.225
**sEEL**	-	-	-	0.00019	1.588	0.093
**m=1**	0.00334	1.532	0.234	0.00464	1.512	0.214
**Higher Order**	0.00037	2.433	0.781	0.00073	2.603	0.826
**Substrate**	0.00647	21.316	7.548	0.00687	21.051	8.022
**Substrate**	0.00390	22.048	2.675	0.00397	21.994	2.763
**Substrate**	0.00255	37.576	14.591	0.00246	38.473	13.499
**Substrate**	0.00136	8.045	5.080	0.00157	7.367	4.572

Supplementary Table S5. Fitting parameters for horizontal m=2 rod for laser off and laser on spectra.

	Laser Off			Laser On		
	**Amplitude (eV^{-1})**	**Center (eV)**	**HWHM (eV)**	**Amplitude (eV^{-1})**	**Center (eV)**	**HWHM (eV)**
**sEEG**	-	-	-	-	-	-
**Thermal**	0.00130	-0.904	0.159	0.00108	-0.893	0.175
**Thermal**	0.00279	0.853	0.214	0.00260	0.858	0.235
**sEEL**	-	-	-	-	-	-
**m=2**	0.00464	1.660	0.177	0.00491	1.651	0.194
**Higher Order**	0.00145	2.349	0.416	0.00097	2.376	0.445
	0.00025	4.217	1.078	0.00035	3.795	0.970
**Substrate**	0.00713	21.200	8.495	0.00681	21.259	8.515
**Substrate**	0.00445	22.208	2.767	0.00439	22.075	2.757
**Substrate**	0.00304	39.864	14.238	0.00259	39.702	13.818
**Substrate**	0.00121	6.600	4.248	0.00118	7.260	4.367

Supplementary Table S6. Fitting parameters for vertical m=2 rod for laser off and laser on spectra.

	Laser Off			Laser On		
	**Amplitude (eV^{-1})**	**Center (eV)**	**HWHM (eV)**	**Amplitude (eV^{-1})**	**Center (eV)**	**HWHM (eV)**
**sEEG**	**-**	**-**	**-**	0.00037	-1.572	0.115
**Thermal**	0.00072	-0.830	0.103	0.00101	-0.906	0.110
**Thermal**	0.00130	0.882	0.232	0.00319	0.837	0.253
**sEEL**	-	-	-	0.00037	1.588	0.115
**m=2**	0.00665	1.540	0.165	0.00387	1.501	0.176
**Higher Order**	0.00191	2.464	0.382	0.00105	2.315	0.418
	0.00097	4.212	1.155	0.00046	3.789	1.041
**Substrate**	0.00726	20.702	8.420	0.00660	21.052	8.178
**Substrate**	0.00391	22.169	2.624	0.00408	21.992	2.812
**Substrate**	0.00283	38.622	13.930	0.00237	38.811	13.327
**Substrate**	0.00193	6.945	3.880	0.00137	7.645	4.268

Supplementary Table S7. Fitting parameters for horizontal m=3 rod for laser off and laser on spectra.

	Laser Off			Laser On		
	**Amplitude (eV^{-1})**	**Center (eV)**	**HWHM (eV)**	**Amplitude (eV^{-1})**	**Center (eV)**	**HWHM (eV)**
**sEEG**	-	-	-	0.00044	-1.572	0.112
**Thermal**	0.00139	-0.773	0.193	0.00145	-0.758	0.202
**Thermal**	0.00145	1.108	0.107	0.00273	1.090	0.107
**sEEL**	-	-	-	0.00044	1.572	0.112
**m=3**	0.00400	1.493	0.153	0.00408	1.472	0.165
**Higher Order**	0.00145	2.170	0.574	0.00132	2.243	0.615
	0.00036	4.291	1.270	0.00052	3.942	1.143
**Substrate**	0.00669	21.386	7.663	0.00642	21.370	7.957
**Substrate**	0.00397	22.099	2.669	0.00415	21.999	2.768
**Substrate**	0.00273	38.352	14.151	0.00256	38.581	14.311
**Substrate**	0.00142	8.332	4.861	0.00139	8.562	4.469

Supplementary Table S8. Fitting parameters for vertical m=3 rod for laser off and laser on spectra.

	Laser Off			Laser On		
	**Amplitude (eV^{-1})**	**Center (eV)**	**HWHM (eV)**	**Amplitude (eV^{-1})**	**Center (eV)**	**HWHM (eV)**
**sEEG**	-	-	-	0.00091	-1.572	0.078
**Thermal**	0.00199	-0.693	0.160	0.00262	-0.702	0.176
**Thermal**	0.00651	0.616	0.147	0.00519	0.610	0.159
**m=2**	0.00288	1.031	0.191	0.00359	1.001	0.210
**m=3**	0.00429	1.457	0.115	0.00343	1.460	0.126
**sEEL**	-	-	-	0.00091	1.572	0.078
**m=4**	0.00091	1.723	0.181	0.00084	1.681	0.199
**Higher Order**	0.00337	2.322	0.533	0.00220	2.244	0.492
	0.00166	4.358	1.198	0.00085	3.986	1.083
**SiN**	0.00189	7.789	3.916	0.00138	7.958	4.148
**SiN**	0.00849	21.047	8.227	0.00674	21.557	8.264
**SiN**	0.00378	22.476	2.676	0.00411	22.370	2.901
**SiN**	0.00350	39.559	14.665	0.00262	39.786	14.249

SI – DDA and e-DDA

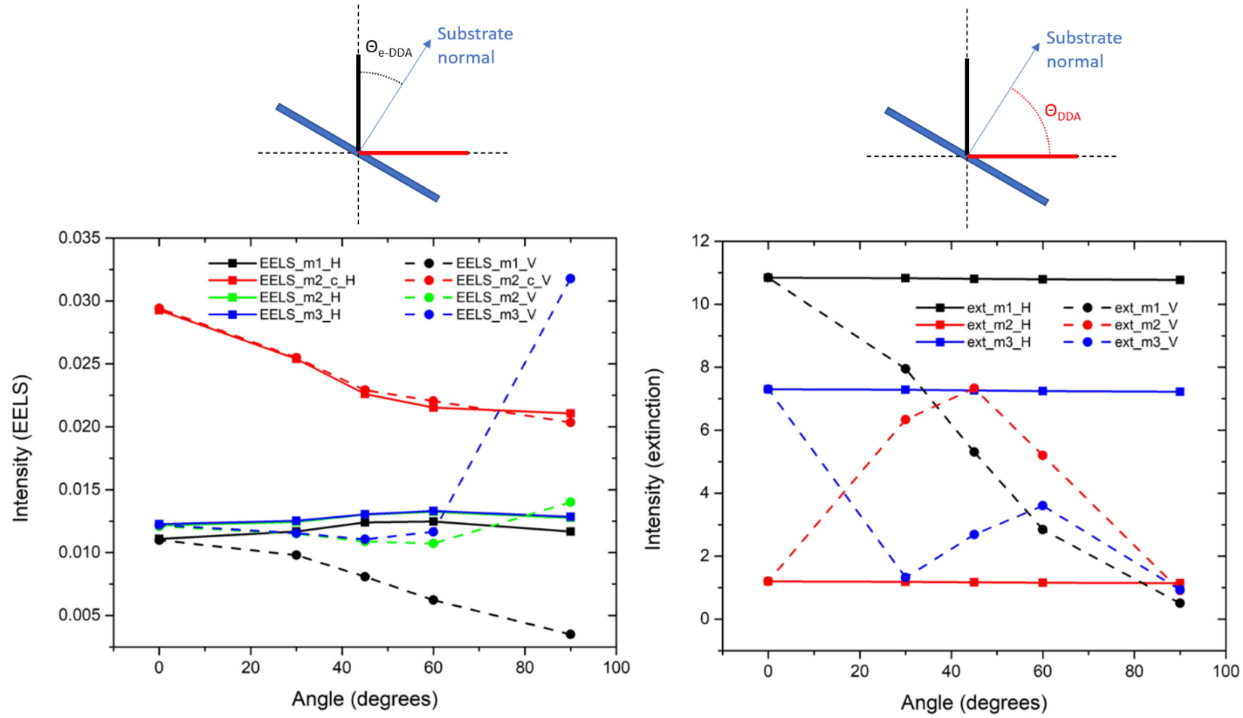

Supplementary Figure S4. a) e-DDA spectra for different electron/substrate angles and b) DDA spectra for different wave-vector/substrate angles.

References

1. Liu, C. *et al.* Continuous Wave Resonant Photon Stimulated Electron Energy-Gain and Electron Energy-Loss Spectroscopy of Individual Plasmonic Nanoparticles. *ACS Photonics* (2019). doi:10.1021/acsphotonics.9b00830
